# Influence of *Cryptosporidium parvum* and *Giardia duodenalis* on glucose transport mechanisms and tight junctions in co-infected enterocytes

**DOI:** 10.1186/s13071-026-07537-4

**Published:** 2026-06-25

**Authors:** Cora Delling, Manuela Kirchner, Katharina May, Franziska Dengler

**Affiliations:** 1 Leipzig University, Leipzig, Germany; 2 University of Hohenheim, Stuttgart, Germany

**Keywords:** *Cryptosporidium parvum*, *Giardia duodenalis*, *Giardia lamblia*, *Giardia intestinalis*, Co-infection, Glucose transport, Tight junctions

## Abstract

**Background:**

*Cryptosporidium parvum* and *Giardia duodenalis* (assemblages A and B) are ubiquitously occurring protozoan parasites infecting a broad range of hosts. Co-infection with both parasites in suitable hosts have been reported, but information on structural or functional alterations in host cells caused by simultaneous infection is rare. Previous findings showing an enhanced replication of *G. duodenalis* during co-infection suggest synergistic effects of both parasites that were investigated in this in vitro study.

**Methods:**

The tight junction proteins claudin (*CLDN) 1, 4, 6,* and* 7* as well as the glucose transporters (*GLUT) 1* and *2* of IPEC-J-2 cells were examined comparing single and co-infections on gene expression level after 24 h, 48 h, and 72 h post infection (p.i.). Additionally, an analysis of intracellular glucose levels was performed 48 h p.i.

**Results:**

No significant changes of the gene expression of the examined tight junction proteins were observed. Regarding the glucose transporters, *GLUT2* was significantly decreased in cells infected by *C. parvum* sporozoites compared to cells infected by *G. duodenalis* trophozoites 48 h p.i. (*p* = 0.017) as well as compared to uninfected control cells (*p* = 0.021). Additionally, co-infected cells showed a significantly increased intracellular glucose level (*p* = 0.022) and *C. parvum* infected cells a non-significant trend of an increased intracellular glucose level (*p *= 0.056) in comparison to control cells. Compared to *G*. *duodenalis* mono-infected cells, co-infected cells showed a tendency for higher intracellular glucose levels (*p* = 0.057).

**Conclusion:**

*Cryptosporidium parvum* had an impact on *GLUT2* transcript abundance and also increased glucose levels in mono- and co-infection under the tested conditions, while *G. duodenalis* did not alter the examined glucose transporter and tight junctions markers in this model.

**Graphical Abstract:**

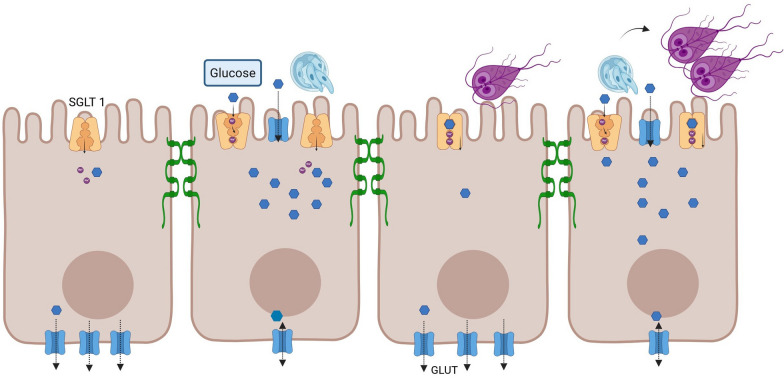

**Supplementary Information:**

The online version contains supplementary material available at 10.1186/s13071-026-07537-4.

## Background

*Cryptosporidium parvum* is an intracellular protozoan parasite causing intestinal disorders in several host species including humans. The parasite is one of the main pathogens associated with moderate to severe diarrhoea and an increased risk of mortality in toddlers from low-income countries [1–3]. The extracellular protozoan parasite *Giardia duodenalis* can be classified in eight assemblages, whereby the assemblages C-H are considered to be rather host specific. Parasites of the assemblages A and B occur in a wide host range and are also zoonotic [4]. The clinical outcome associated with giardiasis ranges from asymptomatic patients to patients with clinical symptoms such as abdominal pain and diarrhoea, whereby the reasons for this variation are poorly understood yet [5].

Although occupying different locations within the intestinal tract of the hosts, both parasites affect the main functions of the intestinal epithelium: shielding the organism from luminal toxins, bacteria and other harmful substances and at the same time mediating the effective vectorial transport of nutrients and electrolytes [6]. Several studies reported the modification of barrier forming tight junction proteins, e.g., claudins, in response to infection with *C. parvum* or *G. duodenalis* in different in vitro and in vivo models [7, 8]. Furthermore, it has been shown that parasites are capable of influencing host organisms on their own behalf for nutrient supply or longevity, including the glucose uptake into the host cells [9–11]. The transporters ensuring the transepithelial glucose transport across the small intestinal epithelium are the Na^+^/glucose cotransporter (SGLT1) and glucose transporter (GLUT) 2. While SGLT1 is located in the brush border membrane and mediates the secondary active uptake of glucose from the gut lumen, GLUT2 is generally found in the basolateral membrane and mediates the facilitated diffusion of hexoses into the bloodstream [12–14]. Additionally, GLUT1 is also able to transport monosaccharides and commonly found in the majority of mammalian cells [15]. Since *C. parvum* lacks many metabolic pathways, it heavily relies on glucose obtained from host cells as important carbon source [16]. In previous studies we showed an altered glucose absorption and metabolism both in an intestinal epithelial cell line and calves infected by *C. parvum*, manifesting in increased intracellular glucose levels, modified glucose transporter abundance and enhanced intracellular glucose metabolism [10, 17]. While the main source of energy for *G. duodenalis* is arginine and the parasite induces changes in the arginine metabolism of the host, *Giardia* can also use glucose for energy supply [18]. Further, it has been shown that *G*. *duodenalis* sonicates caused an enhanced glucose uptake via SGLT1 in Caco-2 cells in presence of high glucose media, maybe showing a potential cell rescue mechanism in response to parasite-induced cell apoptosis [19].

Co-infections with both parasites have been reported in several host species such as calves, dogs, red foxes, and raccoon dogs [20–22]. Therefore, a potentiation of both parasites’ effects on intestinal barrier function as well as glucose transport is possible and may contribute to more severe disease. Nevertheless, information on interactions between the individual pathogens or host cells in parasitic co-infections associated with cryptosporidiosis or giardiasis is rare. To examine the impact on the replication of individual parasites during simultaneous infection, we established an in vitro model in IPEC-J2 cells that we used for *C. parvum* infection solely before [10]. While co-infection led to a significantly increased proliferation of *G. duodenalis* trophozoites, no effect on the growth of *C. parvum* was detected [23]. Based on our findings in former studies [10, 17, 24], we used our in vitro model to investigate the potential influence of co-infections on the nutrient supply and epithelial barrier function. Therefore, we examined glucose transporters and tight junctions comparing single and co-infections with *C. parvum* and *G. duodenalis* in IPEC-J2 cells.

## Methods

For all experiments, trophozoites of *G. duodenalis* WB6 (assemblage AI) obtained from the Robert Koch Institute (Berlin, Germany), and oocysts of an in-house isolate of *C. parvum* (LE-23-Cp-23, Markranstädt) were used as described by Kirchner et al. [23]. The trophozoites were maintained under anaerobic conditions in sterile filtered modified TYI-S-33 medium as stated elsewhere [23]. The infection of IPEC-J2 cells by either *C. parvum* sporozoites or *G. duodenalis* trophozoites or both parasites was realized as specified by Kirchner et al. [23]. More precisely, 2 × 10^5^ IPEC-J2 cells were seeded in 24-well plates and grown for 72 h covered with growth medium (GM) containing Iscove’s Modified Dulbecco´s Medium (IMDM; Gibco™, ThermoFisher Scientific, Waltham, USA), supplemented with 50% Ham´s F-12 Nutrient Mixture W/ GlutaMAX^™^-I (F-12; Gibco^™^, ThermoFisher Scientific, Waltham, USA), as well as 10% foetal calf serum (FCS; PAN Biotech^™^, Aidenbach, Germany), and 0.002 mM L-glutamine (Gibco^™^, ThermoFisher Scientific, Waltham, USA). Further on, 0.5 U/mL Penicillin, 0.5 µg/mL Streptomycin (Gibco^™^, ThermoFisher Scientific, Waltham, USA) and 0.025 mL Amphotericin B (PAN Biotech^™^, Aidenbach, Germany) were added. For infection, excystation of *C. parvum* oocysts was performed as stated elsewhere [23], and sporozoites were resuspended in Dulbecco`s Modified Eagle Medium (DMEM; Gibco^™^, ThermoFisher Scientific, Waltham, USA) containing additionally 2% FCS, 0.5 U/mL Penicillin, 0.5 µg/mL Streptomycin (Gibco^™^, ThermoFisher Scientific, Waltham, USA), 0.025 mL Amphotericin B (PAN Biotech^™^, Aidenbach, Germany), 0.01 mM sodium pyruvate (ThermoFisher Scientific, Waltham, USA), as well as 0.04 mM L-glutamine (Gibco™, ThermoFisher Scientific, Waltham, USA), and added to IPEC-J2 cells washed with phosphate buffered saline three times before (PBS; Gibco^™^, ThermoFisher Scientific, Waltham, USA). In case of *C. parvum* mono-infection, medium was changed after 3.5 h and cells were recovered with GM. For infection with *G. duodenalis* trophozoites solely, IPEC-J2 cells were covered with GM supplemented with 10% modified TYI-S-33. For co-infection, cells were first infected with *C. parvum* as described, and *G. duodenalis* trophozoites in GM with 10% modified TYI-S-33 were added to *C. parvum* infected cells after an incubation time of 3.5 h. In contrast to our former work, in this study only an infection dose of 8 × 10^5^ and 5 × 10^4^ was used for *C. parvum* sporozoites and *G. duodenalis* trophozoites, respectively. Infected cells as well as uninfected control cells were harvested after 24 h, 48 h, and 72 h p.i. Monolayer integrity was microscopically checked carefully before harvesting the cells.

The infection was performed five times (*n* = 5) in case of harvesting after 48 h (*n *= 5), or six times regarding the harvest after 24 h and 72 h (*n *= 6).

To determine changes in gene expression of *GLUT1*, *GLUT2*, *CLDN1*, *CLDN4*, *CLDN6*, and *CLDN7*, two wells of a 24-well plate each were pooled and total RNA was extracted using the ReliaPrep^™^ RNA Cell Miniprep System (Promega GmbH, Mannheim Germany) according to the manufacturer’s instructions including DNAse treatment. One µg of the resulting high-quality RNA was then used to synthesize cDNA using the GoScript^®^ Reverse Transcription System (Promega GmbH, Mannheim Germany) and perform a qPCR reaction with the GoTaq DNA Polymerase kit (Promega GmbH, Mannheim Germany) and the primers as detailed in Table 1 and described previously [10]. Ribosomal protein L4 (*RPL4*) and tyrosine 3-monooxygenase/tryptophan 5-monooxygenase activation protein zeta (*YWAHZ*) were used as reference genes after confirming their stability using RefFinder and the analysis was performed with the ΔΔCt method [25].

**Table 1:** Primers for qPCR

Gene	GenBank accession number	Amplicon size (bp)	Sequence (5′–3′)
Ribosomal protein L4* (RPL4)*	XM_005659862.3	92	F: GCACCACGCAAGAAGATTCA
			R: TGTCTTTGCATACGGGTTTAGC
Tyrosine 3-monooxygenase/tryptophan 5-monooxygenase activation protein zeta* (YWAHZ)*	NM_001315726.1	87	F: GGCCCTTAACTTCTCTGTGTT
			R: GGCTTCATCAAATGCTGTCT
Glucose transporter 1* (GLUT1)*	XM_021096908	140	GCTTCCAGTATGTGGAGCAAC
			GGAAGCCGGAAGCAATCTCA
Glucose transporter 2* (GLUT2)*	NM_001097447	91	F: TGCTCCAACCAAGTTCAGGG
			R: AGTCGAGGCCTATGATCTGAC
Claudin 1* (CLDN1)*	NM_001244539.1	90	F: CCCCAGTCAATGCCAGATATGA
			R: CAAAGTAGGGCACCTCCCAG
Claudin 4* (CLDN4)*	NM_001161637.2	148	F: CGCGACTTCTACAATCCCCT
			R: GAGTAGGGCTTGTCGGTACG
Claudin 6* (CLDN6)*	NM_001161645.1	181	F: ATCGTCCTGACGCTGTTTGG
			R: AGCAGCGAGTCATACACCTT
Claudin 7* (CLDN7)*	NM_001160076.1	115	F: TCATCGTGGCAGGTCTTTGT
			R: GGCAGGGCCAAACTCATACT

To analyse the intracellular glucose levels of control, single infected and co-infected cells at 48 h p.i., the Glucose-Glo^™^ Assay (Promega GmbH, Mannheim, Germany) was used as described before [10]. First, the monolayers were checked for integrity microscopically, then placed on ice and washed with ice-cold PBS. Following the manufacturer’s instructions, cells were inactivated first using a 2:1 mixture of PBS and inactivation solution (stock solution: 0.6 N HCl), and neutralized afterwards (stock solution: 1 M Tris; 3:1). After incubation with Glucose Detection Reagent for 1 h, luminescence was recorded using a GloMax^®^ 96 Microplate Luminometer (Promega GmbH, Mannheim, Germany). The experiment was performed four times (*n* = 4).

Furthermore, immunofluorescent staining for SGLT1 was performed in representative wells of one infection time point each. Therefore, cells were fixed in 4% formaldehyde, preincubated with 4% goat serum and incubated with rabbit-anti-SGLT1 (Bioss Antibodies, #bs-1128R, 1:100) and fluorescently labelled goat-anti-rabbit IgG antibody (Thermo Fisher Scientific, # A-21206, 1:200). In parallel cell nuclei were counterstained with DAPI (Sigma Aldrich, Munich, Germany, 1:5,000) before mounting with glycerol gelatin (Sigma Aldrich, Munich, Germany). The staining was documented using a Leica DM IL LED Fluo IVD microscope and the Leica Application Suite X Software version 3.5.1 using identical settings (exposure time, gain) for each picture.

Graphs were created using GraphPad Prism 11.0 (GraphPad software Inc., San Diego, USA). Statistical analysis was performed using SigmaPlot 14.5 (Systat Software Inc., Erkrath, Germany). A Shapiro–Wilk test was performed to check for normality of the data. Statistical differences between two or more groups were tested using repeated measures one-way analysis of variance (ANOVA) with a subsequent Holm–Sidak test as appropriate. We performed several independent experiments for each analysis, which were considered as repeated measurements and analysed separately for each time point. Statistically significant differences were assumed at *p *< 0.05.

## Results

**Fig. 1:**
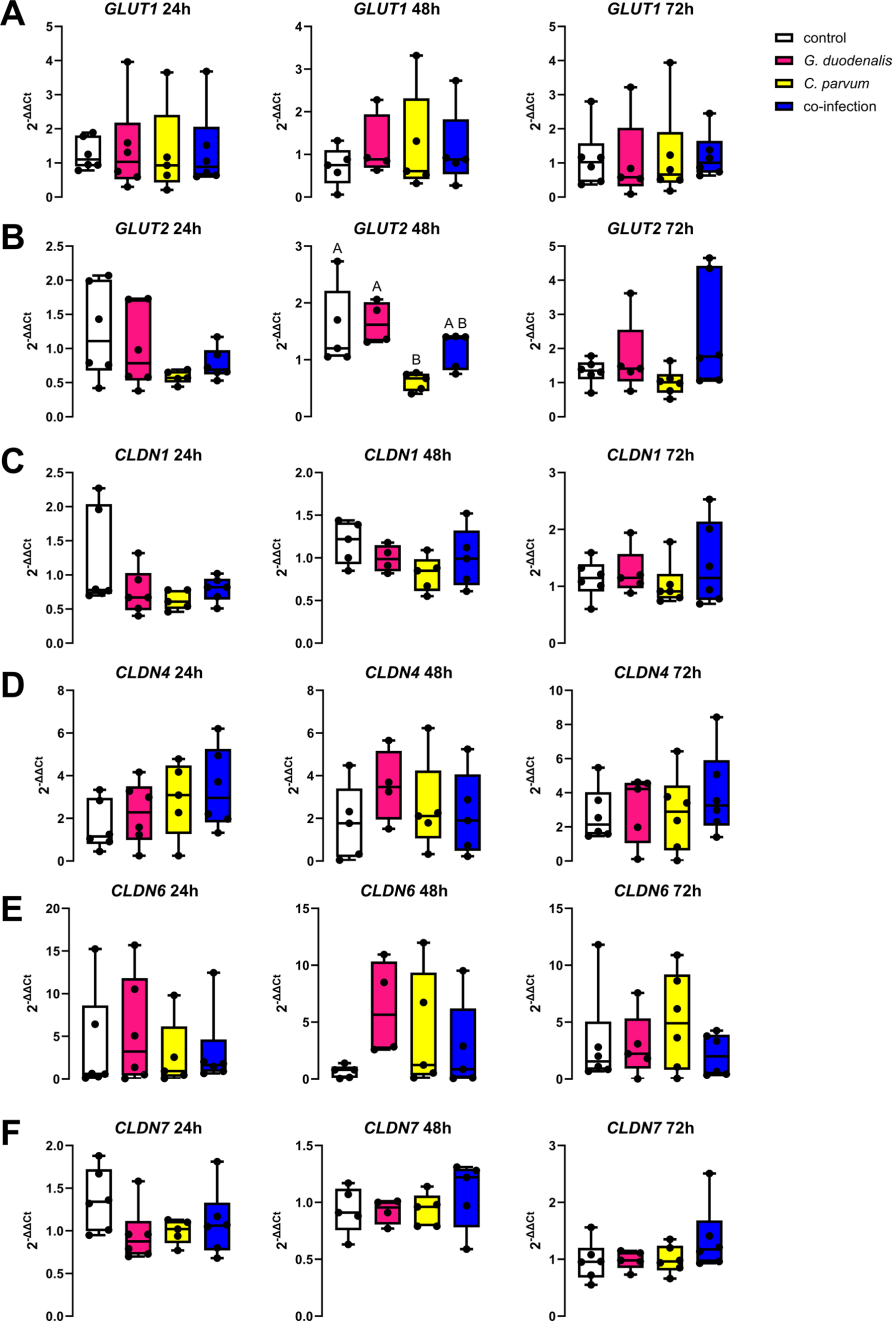
mRNA expression of glucose transporters and tight junction genes of control cells, mono-infected cells and co-infected cells at 24 h, 48 h, and 72 h p.i.: **A** *GLUT1*, **B** *GLUT2*, **C** *CLDN1*, **D** *CLDN4*, **E** *CLDN6*, **F** *CLDN7*. The boxes show median and 25th/75th percentiles, whiskers show minimum and maximum values of the data set, dots represent replicates of independent experiments; *n* = 5 (48 h), *n* = 6 (24 h, 72 h); different letters within the graphs (**A**, **B)** indicate significant differences between the treatment groups, *p* < 0.05; one-way RM ANOVA with post hoc Holm–Sidak test

The mRNA expression of *GLUT1* was not significantly altered between the infection groups and control cells at any time point p.i. (Fig. 1A). In contrast, the mRNA expression of *GLUT2* was significantly decreased in cells infected by *C. parvum* compared to cells infected with *G. duodenalis* (one-way RM ANOVA with post hoc Holm–Sidak test, *p* = 0.017) and compared to uninfected control cells (one-way RM ANOVA with post hoc Holm–Sidak test, *p* = 0.021) at 48 h p.i. (Fig. 1B). The same trend was visible at 24 h and 72 h p.i. (one-way RM ANOVA with post hoc Holm–Sidak test, *p *= 0.062). The mRNA expression level of *CLDN1*, *CLDN4*, *CLDN6*, and *CLDN7* was not significantly altered by mono- or co-infection, but a trend towards a downregulation of *CLDN1* (one-way RM ANOVA with post hoc Holm–Sidak test, *p* = 0.073) and *CLDN7* (one-way RM ANOVA with post hoc Holm–Sidak test, *p* = 0.084) in infected cells was observed 48 h and 24 h p.i., respectively (Fig. 1C–F).

**Fig. 2:**
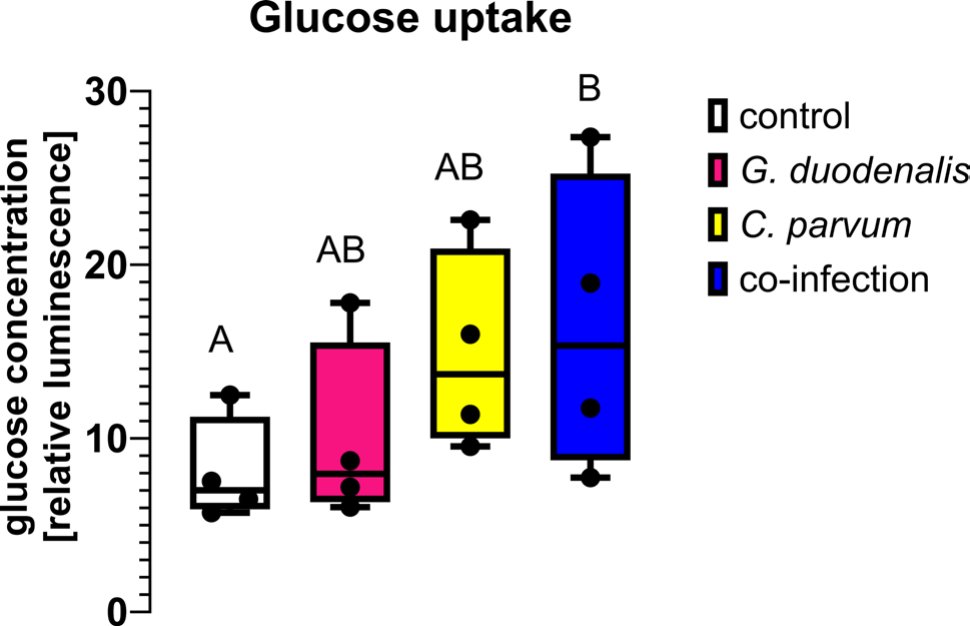
Intracellular glucose level in control cells, mono-infected cells and co-infected cells at 48 h p.i. detected with a luminescent assay; the boxes show median and 25th/75th percentiles, whiskers show minimum and maximum values of the data set, dots represent replicates of independent experiments; n = 4; different letters (**A**, **B**) indicate significant differences between control and co-infection group, *p* < 0.05; one-way RM ANOVA with post hoc Holm–Sidak test

The intracellular glucose level in co-infected cells was significantly increased compared to control cells (one-way RM ANOVA with post hoc Holm–Sidak test, *p* = 0.022) and showed a tendency toward an increase compared to *G. duodenalis* solely infected cells (one-way RM ANOVA with post hoc Holm–Sidak test, *p* = 0.057) (Fig. 2). Furthermore, *C. parvum* mono-infected cells showed a non-significant trend towards an increased intracellular glucose level compared to control cells (one-way RM ANOVA with post hoc Holm–Sidak test, *p* = 0.056) (Fig. 2).

*SGLT1* expression levels were too low to be detected on mRNA level, but immunofluorescent staining of the protein revealed a positive staining in the cell membranes, with a tendency of the membrane signal to increase in the *C. parvum* mono-infection compared to the other experimental groups (Additional file 1).

## Discussion

Based on our previous finding of enhanced proliferation of *G. duodenalis* in the co-infection model with *C. parvum*, we hypothesized that the co-infection has additive effects enhancing the uptake of glucose into infected cells benefitting both parasites [23]. However, the results of this study clearly show a main effect of *C. parvum* on glucose uptake mechanisms, confirming previous data regarding the influence of *C. parvum* infection on glucose uptake into enterocytes [10], but do not support additive effects of the co-infection.

Although *GLUT2* was downregulated on mRNA level in our current and previous studies, a dislocation of the protein from the membrane was observed in the IPEC-J2 model [10], potentially mirroring a recruitment of the transporter to the apical membrane that could be demonstrated in vivo [17]. Therefore, the mRNA expression may be opposed to the activity of the transporter, possibly in the course of a beginning counterregulation. With GLUT2 mediating the uptake of glucose along a concentration gradient, which can be assumed to exist in the cell culture model using a high glucose cultivation medium, this explains the increased intracellular glucose concentrations that were measured in both *C. parvum* and co-infected cells and underline the findings on mRNA level that mono-infection with *G. duodenalis* has no effect on glucose transporter expression also on the functional level.

In our previous studies we additionally found increased gene expression levels and functionality of SGLT1 in *C. parvum* infected enterocytes as well [10, 17]. The immunofluorescent stainings performed in this study hint on an enhanced signal for SGLT1 in the membrane of *C. parvum* infected cells, but again no effects of *G. duodenalis* or co-infection were observed, emphasizing the specific effect of *C. parvum* on glucose transport. This difference in protein localization was not visible in our previous study using the same cell line and might be due to modifications of the protocol regarding the use of different parasites stages for infection, precisely oocysts versus sporozoites. Furthermore, the cells may have been less differentiated due to a different passage compared to the previous study. This could also be a reason why, in contrast to former studies, we failed to detect *SGLT1* on mRNA level.

Yu and colleagues [19] demonstrated an increase of SGLT1-mediated glucose uptake and *SGLT1* expression in Caco-2 cells exposed to *G. duodenalis* sonicates for 24 h in the presence of high glucose media. The authors concluded that the enhanced glucose uptake may represent a defence mechanism against parasite-induced cell apoptosis. In contrast, examining *G. lamblia* infection in human duodenum from patients with chronic giardiasis, Troeger et al. [26] showed a reduced activity of SGLT1. Those differences may be linked to different infection models, time points and infection doses examined in several studies among other things. Additionally, the infection dose used in our study did not alter cell viability [23] and might therefore not interfere with cell metabolism or initiate cell apoptosis.

Interestingly, co-infection showed no influence on *C. parvum* growth in vitro, while *G. duodenalis* replication was significantly increased in presence of *C. parvum* [23]. In the light of our recent findings it could be hypothesized that a beneficial effect of the increased intracellular glucose levels (mediated by *C. parvum*) on the viability and replication of *G. duodenalis* exists. Yet there may be other factors benefitting *Giardia* trophozoites which might explain the increased parasite replication in presence of *C. parvum* that need to be elucidated in future studies. *Giardia* trophozoites preferably use the amino acid arginine instead of glucose for ATP generation and were shown to reduce host cells’ proliferation, induce a halt in cell cycle and strain-dependently also apoptosis [18, 27, 28]. On the other hand, it was hypothesized that *C. parvum* is also able to modulate cell apoptosis for its own benefit [29], so one may speculate that the modulation of host cell viability and integrity during co-infection could be related to an enhanced *Giardia* proliferation.

The epithelial integrity and thus resistance to parasitic infection is determined by the barrier forming tight junction proteins. A disruption of several tight junction proteins by *C. parvum* infection has been shown before and might also be linked to the enhanced replication of *G. duodenalis* in the co-infection model. However, we could only find small trends for a decreased mRNA expression of *CLDN1* and *CLDN7* in infected cells.

Consistent with our findings, in Caco-2 cells no changes in mRNA levels were detectable regarding the tight junction protein *CLDN4* despite protein levels were decreased after *C. parvum* infection [7]. In contrast, both mRNA and protein levels of CLDN4 were significantly decreased in C57BL/6 mice infected with *C. parvum* [7]. These differences may be explained by limited replication of *C. parvum* in simpler in vitro models compared to organoids or in vivo models. Also, in Caco-2 cells, Kraft et al. [30] examined tight junction proteins including CLDN1 by immunofluorescence assay after *Giardia* infection and found no significant changes compared to control cells. Based on the outcome, the authors concluded that current in vitro models mirror more likely asymptomatic host–parasite interactions than infections with clinical signs. Regarding giardiasis, Maia-Brigagão et al. [31] could not show changes in the quantity of tight and adherens junctional proteins, but an altered localization of these proteins was observed by immunofluorescence in *Giardia*-infected Caco-2 cells. Contrary to this, Ma’ayeh et al. [8] showed an upregulation of *CLDN4* gene expression in Caco-2 cells after exposure to *G. duodenalis* assemblage B for 1.5 h, 3 h, and 4.5 h. Additionally, the authors also investigated host cells’ transcriptome showing potential effects on tight junctions’ biogenesis and integrity [8]. Furthermore, Troeger et al. [26] showed a downregulation of *CLDN1* in the duodenum of patients with chronic giardiasis. Nevertheless, comparing different studies with varying protocols is difficult and may explain differences in individual study outcomes.

## Conclusion

In conclusion, we used an in vitro co-infection model to examine effects on glucose transport and tight junction proteins by the parasites *C. parvum* and *G. duodenalis*. While influence on intracellular glucose levels and *GLUT2* by single infection with *C. parvum* was replicable and comparable to the co-infection, *G. duodenalis* mono-infection showed no effect in regard to the examined parameters. Thus, one may hypothesize that *G. duodenalis* might benefit from the *C. parvum* mediated intracellular increase in glucose supply in co-infections. Nevertheless, a range of other factors could be responsible for enhanced *Giardia* proliferation and more studies are needed. Quantitative analysis of protein levels was not performed in this study, so further investigations could extend the data collected. The infection doses used in this study were selected, because cell viability was not affected while an enhanced replication of *G. duodenalis* in co-infection compared to control cells was detectable in our previous study [23]. Therefore, the use of higher infection doses and a more complex model, e.g., bovine-derived intestinal organoids as described in previous studies [32], may improve the reflection of clinical disease and associated cellular processes in host cells in a more realistic way and may alleviate limitations such as the restricted life cycle of *C. parvum* in cell culture.

## Supplementary Information

Supplementary Material 1.

## Data Availability

All data are provided within the manuscript.
