# Supplementary figures and images for: Influence of Cryptosporidium parvum and Giardia duodenalis on glucose transport mechanisms and tight junctions in co-infected enterocytes
Source: Parasit Vectors. 2026 Jun 25;19:263. doi: 10.1186/s13071-026-07537-4 (PMC13308484; doi:10.1186/s13071-026-07537-4)

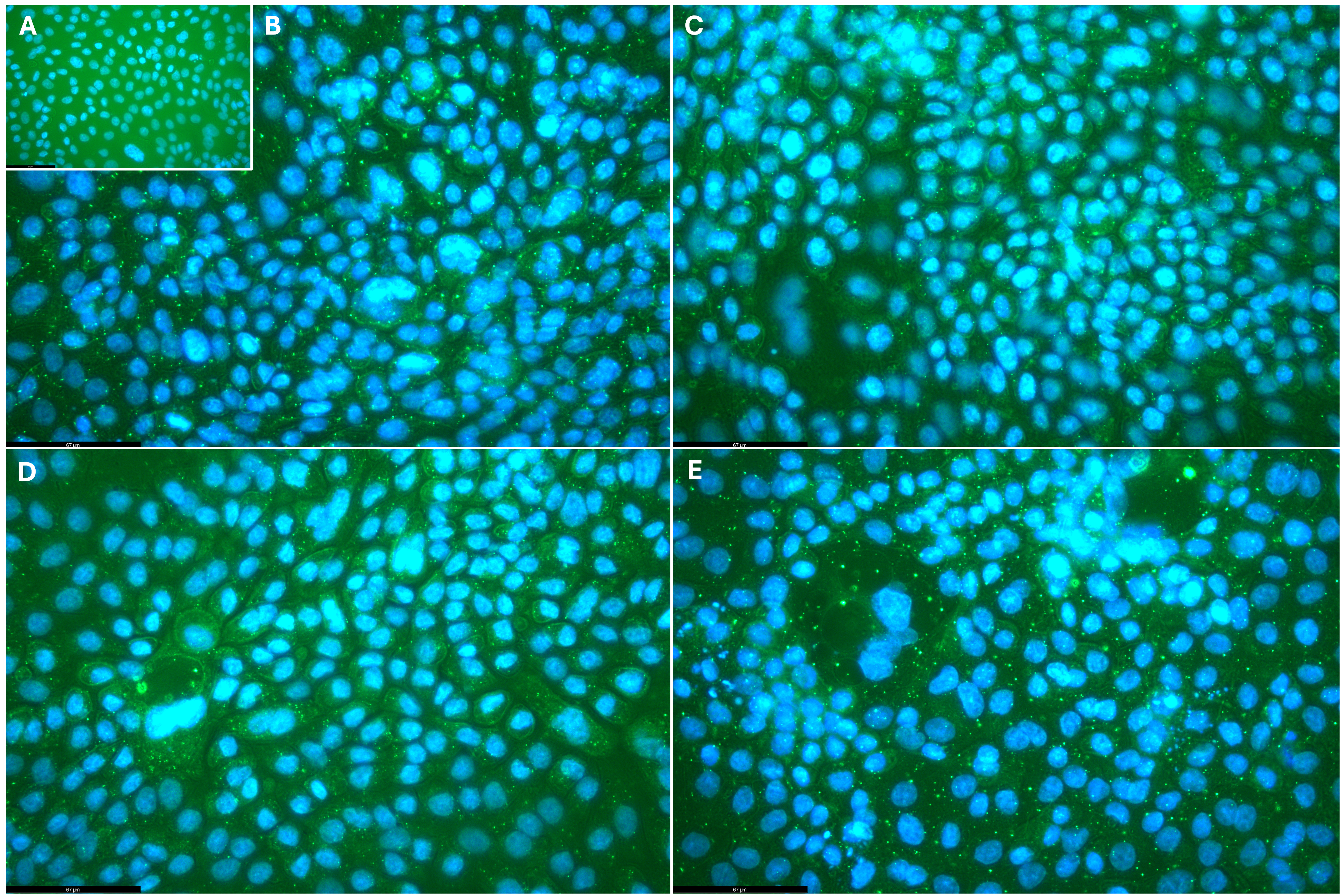

Supplement: Supplementary file 1 — Supplementary Material 1. [file 13071_2026_7537_MOESM1_ESM.tif]
